# The roles of actors in nature-positive transformations

**DOI:** 10.1007/s13280-026-02420-z

**Published:** 2026-06-10

**Authors:** Jennifer Yarnold, Josie Carwardine, Sandra Oliver, Yuwan Malakar, James D. Langston, Ilisapeci Lyons, Anthony O’Grady, Michael Dunlop, Piers Dunstan, Sam Nicol

**Affiliations:** 1 The Commonwealth Scientific and Industrial Research Organisation (CSIRO), GPO Box 2583, Brisbane, Qld 4001 Australia; 2 The Commonwealth Scientific and Industrial Research Organisation (CSIRO), GPO Box 1700, Canberra, ACT 2601 Australia; 3 The Commonwealth Scientific and Industrial Research Organisation (CSIRO), PO Box 12139, Earlville BC Cairns, QLD 4870 Australia; 4 The Commonwealth Scientific and Industrial Research Organisation (CSIRO), Private Bag 12, Hobart, TAS 7001 Australia; 5 The Commonwealth Scientific and Industrial Research Organisation (CSIRO), GPO Box 1538, Hobart, TAS 7001 Australia

**Keywords:** Actors, Biodiversity, Nature positive, Sustainability transitions, Transformation

## Abstract

Nature positive is gaining momentum as a global goal to halt and reverse nature loss. Yet the scale and pace of action are inadequate to curb accelerating biodiversity decline, threatening the foundations of economies and society. Applying sustainability transitions frameworks, we map key system actors to conceptualise their drivers, barriers, interactions and roles they can play to overcome institutional constraints and advance six nature transformation strategies. Our analysis proposes four ideal-type roles for transformative change: (1) *direct impactors* adopt solutions to directly halt or reverse nature’s decline; (2) *rule changers* act to reshape institutional structures and societal values to drive change, (3) *enablers* provide critical supports; and (4) *transformers* challenge regimes through new ideas and solutions. We contend that individuals, organisations and coalitions can fulfil each and even multiple roles depending on actual and perceived agency and that coordinated, multi-actor coalitions across diverse knowledge systems can accelerate nature-positive futures.

****Graphical abstract**:**

**Figure:**
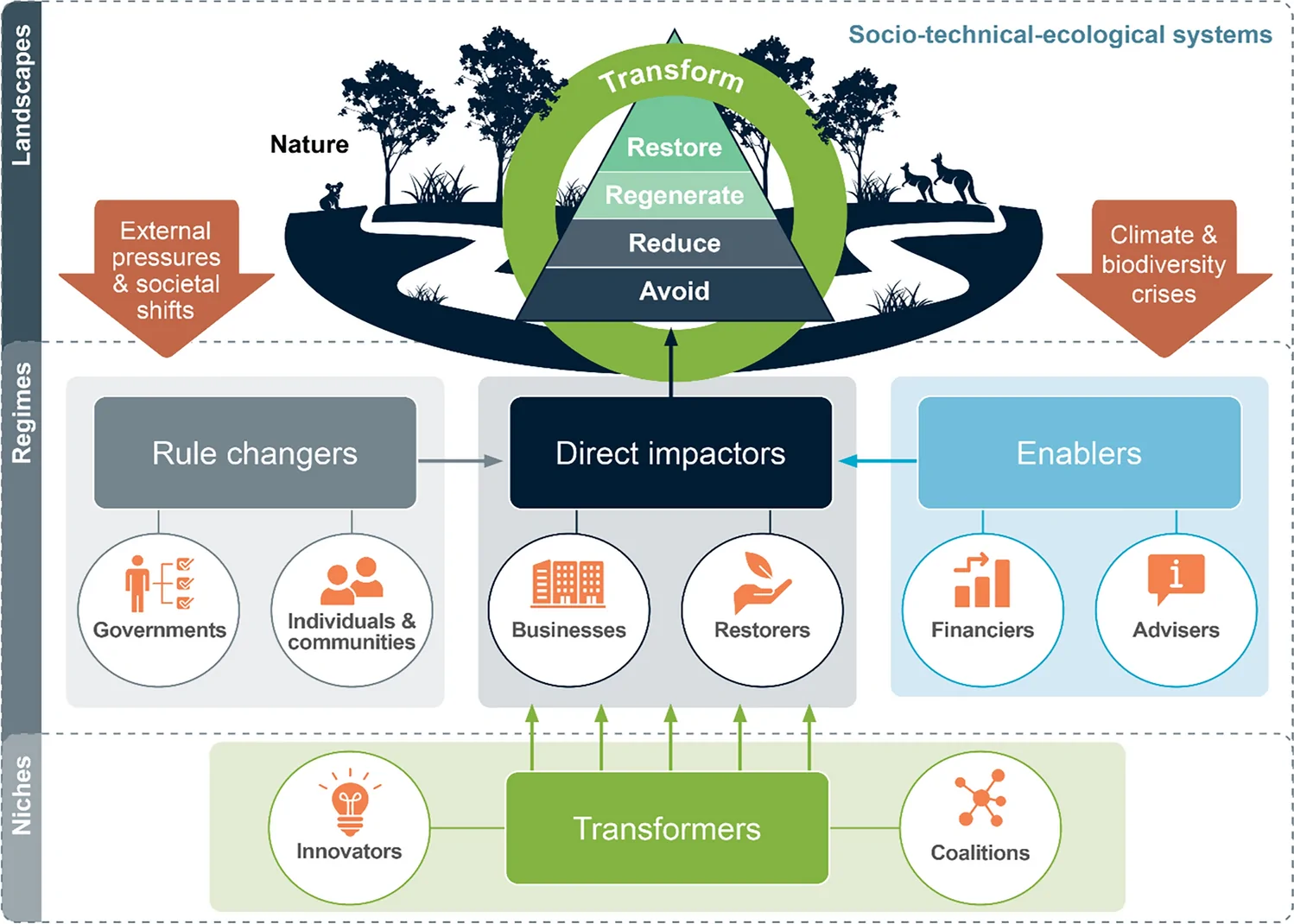

## Introduction

Biodiversity loss, climate change and pollution form the interconnected challenges of the ‘triple planetary crisis’ (UNFCCC 2022). Nature—meaning the living and non-living environmental elements that exist outside direct human control, including biodiversity, ecosystems, landscapes, and the biophysical systems that support them—underpins the cultural vitality, livelihoods and plurality of values that sustain and enrich human wellbeing (Pereira et al. 2020). The World Economic Forum (WEF) estimates that over half of global GDP (~ US$58 trillion) substantially depends on nature directly and indirectly through the provision of raw materials, and the regulating and support services that underpin food and fibre production and resilient supply chains (WEF 2020a). Yet unsustainable modes of production and consumption are driving planetary boundaries to tipping points that threaten ecological collapse (Richardson et al. 2023).

In response, the Nature Positive Initiative was launched by a global coalition of business, finance and conservation organisations. Aligned to the Kunming-Montreal Global Biodiversity Framework (GBF), nature positive is defined as a ‘global goal for nature’ to ‘halt and reverse nature loss by 2030 and set the world on a path towards full ecological recovery by 2050’ (CBD 2022). Nature positive marks a step change to earlier conservation ambitions like ‘no net loss’ that have been applied on a project-by-project basis and typically fail to address the underlying drivers of nature's decline (Maron et al. 2024). Beyond environmental and societal benefits, it is estimated that transitioning to a nature-positive economy could generate more than $10 trillion annually for businesses and create 395 million jobs by 2030 (WEF 2020b).

A 2024 seminal report from the Intergovernmental Science-Policy Platform on Biodiversity and Ecosystem Services (IPBES, referred hereafter as the IPBES Transformative Assessment) asserts that incremental approaches are insufficient to reverse biodiversity loss and its repercussions for human wellbeing. Instead, it calls for a paradigm shift in how humanity interacts with nature—emphasising that collaborative and cooperative contributions from all actors and sectors are paramount to achieving the global goal for nature (IPBES 2024).

However, the cooperative roles and interactions of actors in driving transformative change remain poorly defined (de Haan and Rotmans 2018; Lesch et al. 2023). Addressing these gaps requires deeper insights into the relational dynamics among actors, the drivers and constraints shaping their decisions, and the enabling mechanisms through which coordinated action can scale. Accordingly, our research applies a systems approach to clarify how system actors can interact and align their efforts to overcome barriers and contribute to transformational strategies that can accelerate a systemic shift towards a nature-positive future (IPBES 2024).

Below we provide an overview of sustainability transitions concepts, institutional theories and the multi-level perspective framework (MLP) as they relate to nature transitions (Geels 2010; Avelino and Wittmayer 2016; Loorbach et al. 2017). We apply the MLP framework, systems thinking and rich picture mapping to construct a conceptual actor-network map of a nature-positive systems transformation, defining and describing the primary drivers, roles, barriers, enablers and interactions of actors based on a review of emergent nature-positive transitions literature. We explore the alignment of actors’ roles in advancing six synergistic and transformative strategies for nature outlined in the IPBES Transformative Assessment and how they address key institutional dimensions. We conclude by discussing challenges and opportunities for effective collaboration and perspectives for further research to advance nature-positive transitions.

## Context

Nature-positive transformations can be understood through sustainability transitions concepts which explore how deeply embedded but unsustainable systems can shift towards more sustainable configurations (Markard et al. 2012). Such systems are commonly framed as socio-technical systems to emphasise the interplay between technologies and social structures in transitions of particular sectors—e.g. shifts from degradative to regenerative agrifood systems (Darnhofer 2015), fossil-based to renewable energy systems (Geels et al. 2017), and linear to circular modes of production and consumption (Webster and Pascucci 2024). Other studies apply a socioecological lens to examine the interactions between societies and nature (Ohl et al. 2010). Increasingly, scholars integrate both perspectives into a socio-technical-ecological (STE) system, recognising nature’s fundamental role in human systems (Andersson et al. 2024). The nature-positive agenda exemplifies this shift, representing both a novel paradigm and a suite of solutions aimed at systemic change.

While the terms transitions and transformations are often used interchangeably, they differ by the degree and depth of change: *transitions* involve incremental changes within existing structures, often driven by technological innovation and governance, whereas *transformations* entail more profound shifts in values, institutions, and social relations that redefine the foundations of systems (Hölscher et al. 2018). Over time, successive transitions can culminate into deeper transformations (Colloff et al. 2021).

The Multi-Level Perspective (MLP) is a widely used framework for analysing transitions across three interconnected scales of STE systems: the micro (*niches*), meso (*regimes*), and macro (*landscapes*) (Geels 2002). At the meso-level lie regimes—the focal point of transformations. Regimes are conceptualised as prevalent but unsustainable sub-systems comprised of three interrelated components: actors, institutions and materials. Actors are individuals, organisations or coalitions, typically categorised into social groups who share roles, norms and perceptions, and whose actions are shaped by the institutions of the organisations and society in which they’re embedded but not determined by them. Institutions are the ‘rules of the game’ that influence actors’ behaviour—these include formal rules, such as laws, standards and procedures, and informal rules such as norms and customs. Materials are the tangible things, such as the infrastructures, technologies and the natural environment. The co-evolution of institutions, actors’ interactions and the material aspects in which they are embedded stabilises regimes through reinforcing feedbacks, making them resistant to change (Geels 2004).

At the micro-level, however, are niches which serve as protected spaces for experimentation and learning. Within niches, innovations emerge that offer potential sustainability gains and challenge regimes. Despite their transformative promise, niche innovations often struggle to scale within regimes, due to institutional inertia, infrastructure limitations and resistance from regime actors (Geels 2002).

At the broader macro-level of landscapes, external pressures—such as economic shocks, environmental crises, or political shifts—can destabilise regimes and create windows for change. When niche innovations align with top-down pressures, they may penetrate regimes and shape the trajectory of transitions. The dynamic interplay between system actors, institutions and wider contextual issues affects the pace and scale of transitions and determines whether novel solutions remain marginal or gain sufficient momentum to scale and achieve system transformation (Geels 2004).

## Challenge framing

The IPBES Transformative Assessment identifies six synergistic and complementary strategies that can advance deliberate transformative change to halt and reverse nature’s loss (Table 1). Each strategy requires collective efforts. The complexity of systemic transformation means that no single organisation can be ‘nature positive’ in isolation—change must be enacted through the coordinated activities of diverse actors and resources working in complementary ways (Booth et al. 2024).

**Table 1:** Six transformative strategies identified within the IPBES Transformative Change Assessment Report (IPBES 2024)

IPBES transformative strategy		Shortened in-text citation
Strategy 1	Conserve and regenerate places of value to nature and people	S1: Conserve and regenerate
Strategy 2	Drive systemic change in the sectors most responsible for biodiversity loss and nature’s decline (agriculture, mining, fossil fuels, forestry, fisheries, infrastructure)	S2: Drive sector change
Strategy 3	Transform economic systems for nature and equity	S3: Transform economic systems
Strategy 4	Transform governance systems to be integrated, inclusive, accountable and adaptive	S4: Transform governance systems
Strategy 5	Shift societal views and values to recognise and prioritise fundamental interconnections between humans and nature	S5: Shift societal views
Strategy 6	Knowledge co-creation and collaboration is woven through all strategies to ensure effective knowledge exchange and a commitment to plurality and inclusion	S6: Knowledge co-creation

As inferred through transitions research, advancing these strategies requires not only the integration of new innovations, but a paradigm shift in rules, values and knowledge systems, and a recalibration of economic, social and policy dimensions to decouple societal progress from nature’s decline (Diaz et al. 2019; Leclère et al. 2020; IPBES 2024). An essential part of this paradigm shift is elevating Indigenous Peoples and their knowledge, governance systems, cultural practices, and stewardship—systems that have sustainably managed lands and waters for millennia (IPBES 2024).

Indeed, the institutions underpinning modern regimes are often the structural drivers of environmental decline (IPBES 2024). Innovation studies identify six institutional dimensions that influence transitions: *legitimacy, financial investment, regulatory and policy frameworks, market structures, knowledge and capabilities,* and *equity* (Table 2). When these dimensions are weak or misaligned, they create friction, manifesting as skill shortages, coordination gaps, resistance from actors, inequitable decision-making and misaligned incentives (Hacker and Binz 2021). Pakizer et al. (2023) emphasise that neglecting any dimension can slow transitions or provoke organised opposition, whereas strengthening them can accelerate and stabilise change.

**Table 2:** Institutional dimensions for enabling sustainability transitions and mechanisms to address them (adapted from Hacker and Binz 2021 and Pakizer et al. 2023)

Institutional dimension	Description	Barriers (if not addressed)
Knowledge & capabilities	The creation, diffusion and adoption of new knowledge and capabilities and Indigenous and traditional knowledge systems to increase understanding and capacity of organisations and individuals to adopt, manage and value nature transition processes and technologies	Gaps in skills, coordination and supply chain coordination can stall progress
Legitimacy	The perception that nature-positive transitions and the solutions and processes involved are acceptable, desirable and aligned with societal norms, values and beliefs	Without legitimacy, transitions can face resistance by system actors
Equity	Broad and inclusive representation of different world views and affected social groups, in particular Indigenous Peoples and local communities in all stages of decision-making, planning processes and structures that integrate different values and ensure benefit sharing, agreed fair outcomes and risk management among stakeholders	Can lead to power imbalances, serving interests of powerful actors, leading to opposition and contestation
Financial investment	Mobilisation of resources for beneficial nature outcomes and removal of perverse incentives	High upfront costs to adopt new innovations or processes can deter action unless offset by financial incentives that reflect nature’s values
Regulatory & policy framework	Regulations, standards and reporting frameworks that ensure environmental gains and removal of legacy regulations that create perverse outcomes	Creates an uneven playing field favouring unsustainable organisations and practices
Market structures	Embedding nature into markets, e.g. through subsidies, incentives, quotas, targets, internalising externalities into market prices	Without supportive market structures that value and embed nature, innovations and solutions fail to scale and become mainstream

Institutions evolve through negotiation, reform, policymaking and cultural adaptation in response to emerging pressures. Actor relationships are therefore central to these processes: by developing new roles, adapting existing ones and redefining responsibilities, actors help dismantle institutional barriers to nature-positive transitions (Schweizerhof and Bieling 2024). Intentional governance of these dynamics can steer transitions towards more just, inclusive and ecologically resilient futures.

## Roles of system actors in nature transformations

**Fig. 1:**
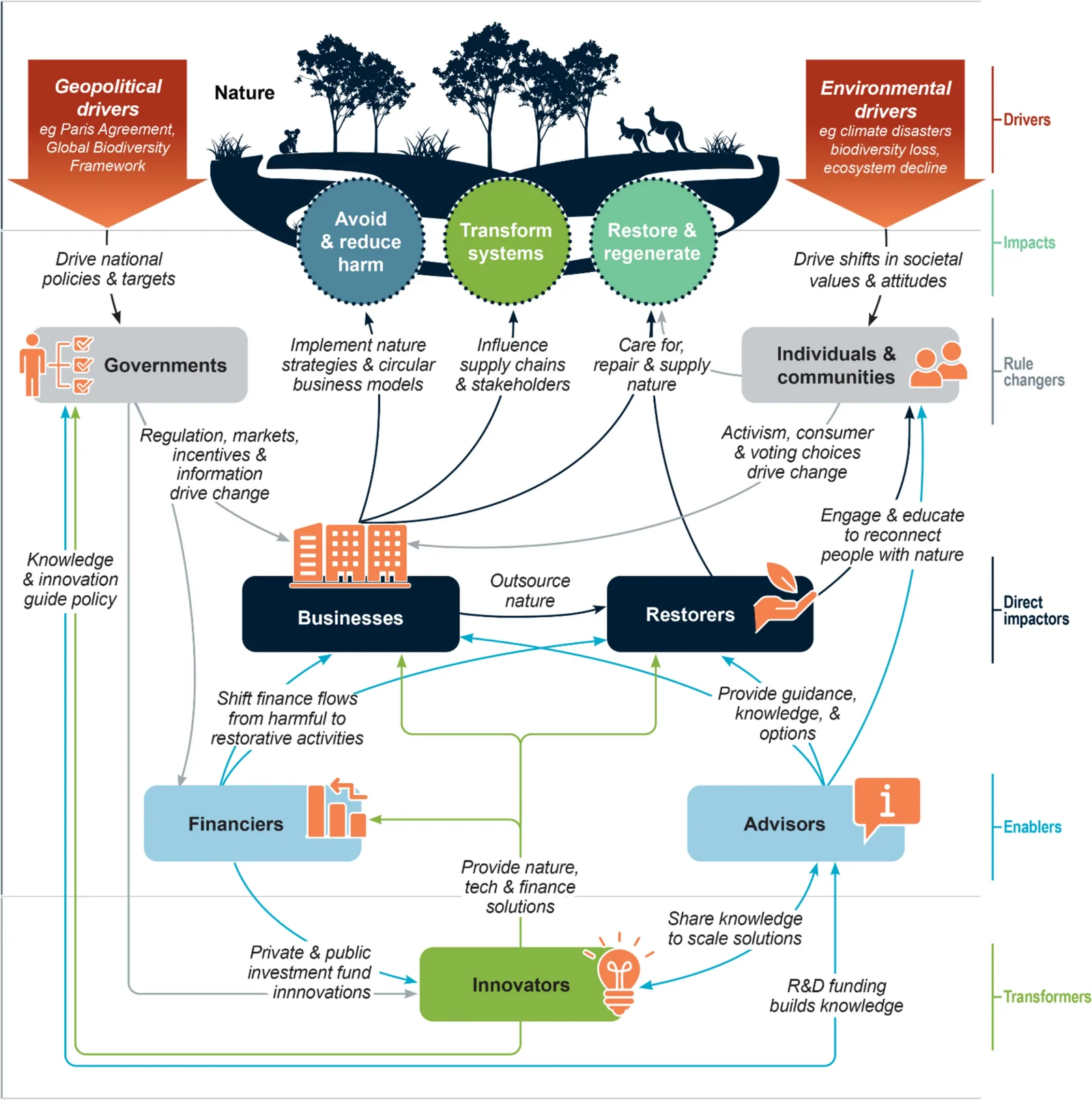
Actor-network map conceptualising the roles and interactions of key actor groups to enable a nature-positive transformation of the current socio-technical-ecological system

Our analysis seeks to identify who are the important actor groups and how can they affect institutional change essential for unlocking new pathways for transformative nature-positive action. To do so, we apply the MLP framework and drawing on emerging nature-positive literature, rich picture mapping (Lewis 1992) and systems thinking (Checkland 1999) we construct an actor-network map (Fig. 1) to conceptualise the dynamic interactions of actors operating across different levels of a socio-technical-ecological system. We identify seven key actor groups—*businesses, restorers, innovators, governments, individuals and communities, financiers* and *advisers*—and describe their motivations to engage in the nature-positive agenda and the roles they can play to overcome institutional barriers (Table 2) and contribute to six IPBES transformative strategies (Table 1) to accelerate nature-positive transformations. We emphasise that the term ‘actor’ is used fluidly to generalise different groups according to a functional perspective of their role in nature transitions, following Fisher and Newig (2016), Loorbach (2017) and de Haan and Rotmans (2018) who emphasise that transition actors are not fixed to one category but can be affiliated to multiple roles and systems.

Figure 1 shows that at the macro-level, landscape pressures are growing due to accelerating rates of environmental degradation and natural disasters causing adverse impacts to biodiversity, human health and wellbeing, food production and the economy. Heightened public concerns of these risks—driven by lived experience, media coverage and mounting scientific consensus—are shifting societal values and attitudes (S5) (WEF 2025)*.* To highlight, a survey across 50 countries found 80% of respondents believe humanity is ‘heading for environmental disaster unless we change our habits quickly’ and call for ‘more government regulation of corporate environmental impacts’—sentiments reflected in growing consumer demand for brands that align with individuals’ values (Ipsos 2024).

In addition to these crises and societal and geopolitical shifts, international organisations and agreements such as the Convention on Biological Diversity and the Kunming-Montreal Global Biodiversity Framework (GBF) are mandating that signatory countries pursue the nature-positive agenda via their obligations under such agreements.

The combination of landscape factors creates cracks to our unsustainable contemporary STE systems, placing pressure on regime actors to take action.

### Innovators

At the niche level, innovators—often driven by environmental concern—learn and develop solutions that enable actors who adopt them to avoid and reduce harms and restore and regenerate nature. Innovations therefore vary widely from nature-based solutions and land restoration approaches to more sustainable technologies and products, circular production processes and business models, governance structures, policy instruments, green finance and market-based mechanisms and likewise innovators can come from a range of sectors (Dimitrova et al. 2013; Seddon et al. 2020). The creation and adoption of innovations contribute to several IPBES transformative strategies including *S1: conserve and regenerate nature*, *S2: drive sector change, S3: transform economic systems*, and *S4: transform governance systems*. Many innovators are driven by passion over profit, however, and initiatives often remain small scale and constrained by a lack of financial investment and limited markets. Scaling innovations at the early stages of development requires financial and other types of support, particularly from *governments* and *financiers*, through, for example, the provision of innovation loans and grants, and protected spaces for experimentation or public procurement to establish markets for green products and services.

### Individuals and communities

At the regime level, shifts in attitudes of individuals and communities can exhibit powerful influence across the system, as the way and extent to which they value, understand and are concerned about nature drives the responses of other actors in the system. As consumers, their demand for sustainable goods and services and corporate sustainability influences businesses’ product designs and activities (supports *S2: drive sector change*); as citizens, their votes shape government policy (supports *S1–4*), and through narratives and advocacy they reshape discourse and society’s relational values with nature thereby boosting legitimacy by normalising nature-positive as a societal goal (Himes and Muraca 2018). Individuals and communities also contribute to nature outcomes directly, e.g. by reducing consumption, recycling, and restoring nature in households and local environments. A special mention is given to individuals who take on the role of *champions*. These influential individuals often catalyse change by initiating and embedding sustainable practices within the organisations they work at, their communities or society (Buhr et al. 2023). Buhr et al. (2023) characterise champions as possessing strong sustainability-oriented beliefs and relevant competencies which enable them to navigate institutional structures and shape policy, organisational and/or societal change through value-driven action and strategic influence. Koistinen et al. (2018) contend that the micro-level agency of champions complements macro-level pressures, supporting new ideas like nature positive to gain traction. By translating sustainability goals into practice and challenging entrenched norms, champions help bridge the gap between innovation and adoption.

A barrier to pro-environmental behaviour among individuals and communities is the growing disconnect from the natural world—what Richardson (2025) terms the ‘extinction of experience’—driven by urbanisation, digital lifestyles, and consumer culture (Borg et al. 2024). Reduced contact with nature diminishes people’s awareness of its benefits and creates psychological barriers such as low perceived behaviour control (i.e. the belief that one’s actions are too insignificant to matter). *Restorers, advisers* and *governments* can all play roles to bridge this disconnect, particularly through the development of local initiatives that encourage public participation in nature-based recreation, restoration and education. Such experiences have been shown to enhance environmental awareness, improve wellbeing and empower people to care for and enjoy their local environments (Ives et al. 2018).

### Governments

Obligated to meet international commitments and responding to societal pressures, the central role of governments is to drive pro-nature behaviour change and phaseout harmful practices (supports *S1–3, S5*). Accordingly, governments are crucial in reshaping many of the formal institutional dimensions needed to create an enabling environment for nature transitions, ensuring that *legal and regulatory frameworks, financial incentives* and *market structures* are fit for purpose. They must apply and remove incentives to ensure actors can improve nature outcomes without competitive disadvantage to drive sectoral change (supports *S2*) (Pakizer et al. 2023). Enabling policy mechanisms span regulatory instruments like nature-based mandates and standards, financial instruments including ecosystem service payments, tax (dis)incentives, and grants, informational instruments such as education and training and market-based instruments, e.g. biodiversity credit schemes. Single instruments are rarely effective however—transformative change requires synergistic policy mixes that evolve through transition phases, coordination between agencies and levels of government, and embedding nature considerations across policy domains, planning and support schemes (Pakizer et al. 2023). Clear policy direction and ambitious targets coupled with public investment are essential to provide certainty for businesses to act and to attract private sector investment (supports *S2* and *S3*). The European Union’s Biodiversity Strategy for 2030 is an example of integrated policy with its Nature Restoration Regulation (2024) that sets ambitious binding targets to restore degraded ecosystems underpinned by biodiversity investments across EU funds to unlock 20 billion euro per year in private and public funding (European Commission 2026).

Governments must navigate entrenched institutional processes, legacy policies, and competing political and economic demands. Because they hold authority over public decision-making, they are central to *transforming governance systems* (S4) to become more integrated, inclusive, accountable, and adaptive. By collaborating with advisers, innovators, communities, and businesses, governments can co-design policy solutions that reflect diverse perspectives and embed societal values of nature and place, helping overcome barriers and enable effective, nature-positive transitions.

### Businesses

Businesses have high leverage to affect nature transitions due to their direct impacts and dependencies on nature and their agility to innovate and respond to community and customer values (Panwar 2023). Accordingly, businesses are the target of many government policies aimed at reversing nature’s decline.

The primary role of businesses is defined in GBF Target 15, which states that ‘businesses assess, disclose and reduce their biodiversity-related risks and negative impacts throughout their operations and supply chains’. To do so, businesses can apply emerging guidance frameworks on nature transitions and mitigation principles (e.g. *avoid, reduce, restore, regenerate and transform*) to adjust their business models, processes, products, services and procurement strategies (SBTN 2020). To achieve net-gains for nature, businesses should go beyond their direct operations to seek ways to restore and improve nature (S1) and drive change across supply chains and sectors (S2). These roles align with niche functions such as experimentation, upscaling and diffusion, where businesses either trial novel nature-positive approaches or use their market position to normalise them within broader regimes.

However, businesses do not play a uniform role in sustainability transitions. Transition studies emphasise that organisations can act as niche actors, regime stabilisers or regime disruptors, depending on their strategic orientation, capabilities and exposure to socioecological risks (de Haan and Rotmans 2018). Often smaller, mission-driven or innovation-oriented firms function as niche pioneers as they have more agency to experiment with new practices, technologies and business models. Others act as early adopters, scaling promising innovations as risks diminish. In contrast, large incumbent firms may simultaneously explore niche innovations while protecting existing profitable models, contributing to uneven transition dynamics. These differentiated roles shape how, and how quickly, nature-positive practices diffuse across sectors.

Motivations for engaging in nature-positive action vary across business types. Some are intrinsically and ethically motivated to reduce their nature-related impacts, such as niche pioneers who act from mission-driven commitments and opportunities to differentiate through innovation (de Haan and Rotmans 2018). Early adopters may be motivated by competitive advantage through reputational positioning as nature-positive expectations become mainstream. Many businesses, however, wait to engage in the nature agenda only through external forces such as regulatory compliance, policy incentives and/or risk mitigation (Liu et al. 2025). Indeed, company boards and institutional investors have a fiduciary duty to address long-term and foreseeable risks to an organisation’s business model—increasingly, these include material nature-related risks, meeting consumer and investor expectations toward sustainability, maintaining social licence—particularly in communities affected by their operations—and avoiding unfavourable media coverage or lobbying against their interests (Liu et al. 2025). Decoupling business productivity from harmful activities can provide benefits of increased resilience and operational efficiency, and access to new markets and investors.

Despite growing motivations to improve their nature position, however, business readiness remains variable. A review of business engagement with biodiversity found institutional barriers include: regulatory uncertainty and a lack of standardisation in reporting, measurement and metrics (*regulatory & policy framework*), limited access to expertise and knowledge (*knowledge and capabilities*), financial constraints or high transaction costs to adjust business processes or purchase new equipment (*financial investment),* a lack of investment-ready conservation projects (*market structure*), and insufficient transparency in biodiversity reporting commitments (*legitimacy*) (Liu et al. 2025)*.* These barriers affect firms differently: smaller innovative firms often face capability and investment constraints, whereas incumbents may experience organisational inertia or conflicting incentives between existing business models and emerging nature-positive expectations. A 2024 survey of Australian business leaders implicated *knowledge and capabilities* as the main impediment—highlighting unclear practical pathways to implement effective change, uncertainty about what nature-positive means in practice, a lack of reliable assessment methods and limited organisational capacity as reasons for inaction (Australian Climate and Biodiversity Foundation 2024). Businesses can overcome these barriers by engaging with other systems actors including advisers, restorers and financiers (Liu et al. 2025). Such collaborations are particularly important for niche actors seeking to scale innovations and for incumbents attempting to integrate nature-positive approaches across complex supply chains.

### Advisers

Advisers are important intermediaries for businesses, governments and other actors. Driven to produce and share knowledge, their role is to build capability and skills across the system by translating knowledge of nature problems and solutions in a way that meets actors’ needs and competencies and to improve decision-making (*S6: knowledge co-creation*) (Wittmayer and Schäpke 2014). Advisers can support businesses by helping them to develop and implement feasible nature transition strategies, identify, adopt and operate niche innovations, and formulate and understand the cost–benefits of integrating nature strategies. Scientific advisers and sustainability consultants, in particular, can help businesses to quantify their nature-related impacts, risks, dependencies and opportunities, set science-based targets, and develop indicators and methods for evaluation (Addison et al. 2019). They also play important roles as boundary spanners and knowledge brokers by connecting diverse stakeholders and knowledge systems to facilitate collaboration and learning to co-produce solutions (Goodrich et al. 2020). By helping businesses adopt sustainable innovations, advisers in turn help innovators scale and deploy their solutions into specific contexts.

### Restorers

The central role of restorers is to protect and improve nature at niche, system and landscape scales (S1). They provide essential support for businesses to achieve net-gains for nature—either as advisers or as delivery partners in restoration projects, particularly for businesses that do not interface with or have direct access to nature. Although avoiding harms is prioritised in the nature mitigation hierarchy, Blignaut et al. (2014) point out that restoration is ‘the only way to replenish the quality and quantity of goods and services that ecosystems can sustainably deliver’. Accordingly, restoration should not be viewed as a ‘luxury item, but rather as something to be integrated in holistic sustainable development thinking at all scales’. Restorers are driven by innate environmental concern—particularly Indigenous land stewards with strong spiritual connection and a long history of sustainable management of land and waters. Where appropriate, governments, businesses, communities and restorers should support Indigenous-led governance and management of Country and centralise Indigenous and local communities’ perspectives and knowledge systems in decision-making (S4)—this is vital to protecting and improving biodiversity of high value to people and to create co-benefits for Indigenous communities (S1) (Burgess et al. 2009).

Many restorers are not-for-profit and rely on government or philanthropic funding, volunteer labour or short-term partnerships with the private sector. However, poor *financial investment* in conservation remains a critical barrier to sustain and scale nature restoration. For example, in 2022, an estimated $5 trillion of global private finance was directed toward activities harmful to nature compared to just $35 billion (less than 1%) for activities that positively impacted nature (UNEP 2023).

### Financiers

To this end, the primary role of financiers is to help system actors overcome *financial investment* barriers by transforming economic systems (S3) so that capital flows of the economy are redirected from harmful activities towards resilience-building and restorative activities. There is increasing interest by financiers in nature investments due to growing market demand, regulatory and reporting requirements, and impact commitments (e.g. SDGs, net zero by 2050 and GBF targets) (Ding and McLaren 2025). However, financiers can be reluctant to invest in nature-related activities due to perceptions of high risk and low returns. Studies estimate that ecosystem conservation and repair provides substantive socio-economic and environmental benefit-to-cost ratios between USD2–40 for each dollar invested (Blignaut et al. 2014; Stewart‐Sinclair et al., 2021). However, nature projects often lack clear revenue streams for investors or depend on indirect benefits or nascent biodiversity markets (Ding and McLaren 2025). This is further hindered by a lack of standardised data and metrics: unlike carbon emissions, biodiversity outcomes are complex, context-specific and take years to generate while financial decisions are dictated by entrenched approaches of calculating risks and returns. Additionally, knowledge gaps between the finance sector (in assessing ecological performance) and by nature-based project developers (in structuring investable proposals) make it difficult to ‘capture’ the value that can be monetised. These barriers are compounded by policy and regulatory inconsistencies which weaken investor confidence (Bromley 2024).

Financial innovations from advisers, innovators and financiers are beginning to rectify some of these challenges, combined with structural shifts in markets, policy and disclosure that seek to align ecological outcomes with financial incentives. Emerging financial products such as green bonds and debt-for-nature swaps provide pathways for generating returns, addressing the barrier of weak revenue streams (Plantinga et al. 2024). Blended finance models for large-scale restoration projects are combining public and/or philanthropic capital to help de-risk early projects for private investors (Barber 2025). Demand for ESG integration—enabled by transparent and standardised disclosure and reporting frameworks (e.g. GRI, ISSB, TNFD)—along with environmental–economic accounting, is helping translate ecological outcomes into financial language, reducing uncertainty and enabling investment comparability for projects and business adopters (Boffo 2020). Nascent biodiversity credit schemes like Australia’s Nature Repair Market are starting to create investable markets for biodiversity and ecosystems. And the insurance sector is realising the investment case of integrating nature-related resilience into risk management and underwriting practices (Insurance Council of Australia 2024).

## A typology of transformative roles

Realising the ambitions of the global goal for nature and enabling a biodiverse future for humanity hinge on the ability of diverse actors to collaborate, innovate and reconfigure entrenched systems. Our actor-network analysis provides a practical lens to understand the roles of actors and how they can strategically engage to overcome institutional barriers of incumbent regimes and support nature transformative strategies within a dynamic and interconnected system. Based on the roles revealed through our actor map, we summarise and cluster actor roles into a high-level typology (Table 3):

1. *Direct impactors,* such as *businesses* and *restorers,* are most able to directly improve outcomes for nature through their activities and operations
2. *Rule changers* influence the actions of other actors by reshaping the formal (*governments*) and informal (*individuals and communities*) institutions of systems
3. *Enablers* such as *financiers* and *advisers* provide critical supports and resources that build capacity and capabilities across the system, and
4. *Transformers,* such as *innovators* and *coalitions* (see section below), actively challenge regimes by creating, implementing and promoting new ideas and solutions.

**Table 3:** Summary of key actors, examples of stakeholders and their respective roles in contributing to IPBES transformative strategies and institutional dimensions for nature-positive transitions

Role classification	Actor group	Stakeholder examples	Central role in nature-positive transformations
Direct impactors	Restorers	Natural resource managers, Landholders,Environmental NGOs, Indigenous people,Communities, Volunteers, Farmers	Conserve and restore ecosystems Strengthen people & nature connections
	Businesses	For-profit organisations in all sectors	Avoid and reduce harms to nature within their operations and across supply chains
Enablers	Financiers	Banks, Asset owners/managers,Insurers, Investment consultants,Fund managers, Donors,Individual investors, Governments,Public finance corporations,Grant bodies	Redirect financial investments away from harmful activities towards low-harm and restorative activities
	Advisers	Researchers, Scientists,Experts, Consultants, Indigenous peoples,Educators, NGOs, Media	Build and broker knowledge, capability and capacity across the system
Rule changers	Governments	National, state, local governments & agencies,Regulators, Policymakers, Independent authorities, Intergovernmental organisations,Auditors	Embed nature and equity across regulatory and policy frameworks, programmes, incentives, and market and governance structures
	Individuals & communities	Citizens, Local communities, Voters,Indigenous peoples and communities,Consumers	Shift societal norms and values through discourse and demand change from governments and businesses via activism, voting and consumer preferences
Transformers	Innovators	Researchers, Entrepreneurs, Start-ups,Indigenous peoples, Businesses, Coalitions,Community groups, Changemakers	Create novel solutions to improve nature outcomes
	Coalitions	Advocacy coalitions, Industry alliances,Place-based community initiatives, Innovation hubs, Knowledge networks	Combine knowledge, skills, future visions and strategies to drive systemic change

Of course, these high-level roles are not fixed—roles can be fluid, dynamic, overlapping and context dependent, reflecting the multifaceted character of agency in transitions. Indeed, actors can contribute to each and multiple roles and may shift between them over time and across contexts. For example, a business may act as a direct impactor by reducing its ecological footprint, an enabler by investing in nature restoration projects, a transformer by introducing a novel product or service, and a rule changer by advocating for policy change. Rather, this simple classification seeks to clarify the functional roles and the actors best placed to undertake them to advance transformative strategies and overcome institutional barriers.

## Roles for coalitions

Transcending the high-level and specific roles of actor groups, coalitions of diverse actors provide a powerful vehicle to accelerate systemic transformations by overcoming equity, legitimacy, skills and capabilities barriers (Ciplet 2022). Wesche et al. (2024) define coalitions for sustainability transitions as ‘groups of different actors who share a common goal for change, and coordinate strategies over time in an effort to shape the socio-technical regime they operate in’. As Bos et al. (2015) ‘no single actor, or group of actors, has the agency and power to create such change on their own’. The co-creation of shared visions through participatory processes that acknowledge different perspectives, in conjunction with combining unique skillsets, experiences, resources, and opportunities, enables these networks of interdependent actors to continually put pressure on regime actors and affect change in different parts of the system (Wesche et al. 2024).

We therefore conceive coalitions as niche-level actors who carve out protected spaces to develop and apply new ways of working beyond the constraints of traditional roles and institutional processes.

## Outlook

From our analysis, three related insights emerge to guide future research:

First, is the critical and relational aspect of agency. Institutional theory underscores a duality: actors are embedded within constraining institutional structures but also possess the capacity to reshape them. Though the multi-level perspective framework infers a kind of temporal sequencing of events for transitions to unfold—i.e. through the development of external landscape pressures and the creation of innovations to spur system actors to act—multiple, simultaneous pathways can advance nature-positive transformations. Feedback between innovations, pressures, actors’ behaviours and institutional change is highly dynamic and intertwined. This means that early adopters and innovators—be they individuals, organisations, or coalitions—can pioneer transitions by demonstrating new ways of thinking and doing, thereby shifting norms and influencing others to act. The theory of planned behaviour infers that agency—a person’s ability to make choices and act on them—stems from their perceived level of control, the subjective norms about what others expect them to do and their attitude towards that behaviour. Here, the dimensions of legitimacy, knowledge and capabilities serve to engender agency in actors to take action by reshaping normative beliefs, value systems and cultural–cognitive rules that underpin behaviour. Future research should focus on ways to strengthen agency within businesses and other actor groups, for example, through the co-creation of support tools and programs that help identify nature-positive futures, solutions and transition plans and demonstrate their implementation through pilot projects and real world case studies.

This follows to our second point that effective collaboration between sectors and actors is imperative for systemic change. Nature-positive transitions require actor’s differing roles, abilities and resources be brought together to overcome barriers and create an enabling environment that facilitates change. Effective collaboration must address frictions that can arise from divergent institutional logics (i.e. profit motives in business versus public mandates in government), power imbalances, mistrust and intellectual property concerns that can hinder coordination and delay action. Transdisciplinary research coalitions can help to break down silos by facilitating diverse stakeholders to experiment and implement inclusive, adaptive and novel governance models, policies, practices and solutions.

Third, elevating and incorporating the diverse ways that people value and relate to nature, particularly recognising Indigenous knowledge systems, are critical for enabling just, holistic and supported nature-positive transitions. Future research implications include exploring and enabling Indigenous leadership and opportunities in nature-positive transitions, including mechanisms for co-creation, and governance that recognise Indigenous sovereignty and epistemologies and support pluralistic pathways to transformation. This is essential for fostering legitimacy, enhancing adaptive capacity and catalysing systemic change aligned with ecological resilience and cultural integrity.

The path to 2030 and beyond will be shaped not only by individual actions but by the collective agency of actor networks. Transformational change can be slow and complex. By clarifying roles, fostering coalitions, and embedding equity and co-design into governance structures, an accelerated transition to a nature-positive future is possible. Ultimately, this shift requires moving from isolated interventions to collaborative, networked and institutionally embedded action. The foundations are in place. The challenge is to build on them faster, deeper and together.
